# Temporal integration of monaural and dichotic frequency modulation

**DOI:** 10.1121/10.0005729

**Published:** 2021-08-03

**Authors:** Katherine N. Palandrani, Eric C. Hoover, Trevor Stavropoulos, Aaron R. Seitz, Sittiprapa Isarangura, Frederick J. Gallun, David A. Eddins

**Affiliations:** 1 Department of Communication Sciences and Disorders, University of Maryland, College Park, Maryland 20742, USA; 2 Brain Game Center, University of California Riverside, Riverside, California 92521, USA; 3 Department of Psychology, University of California Riverside, Riverside, California 92521, USA; 4 Department of Communication Sciences and Disorders, Mahidol University, Phaya Thai, Bangkok 10400, Thailand; 5 Oregon Hearing Research Center, Oregon Health and Science University, Portland, Oregon 97239, USA; 6 Department of Communication Sciences and Disorders, University of South Florida, Tampa, Florida 33620, USA

## Abstract

Frequency modulation (FM) detection at low modulation frequencies is commonly used as an index of temporal fine-structure processing. The present study evaluated the rate of improvement in monaural and dichotic FM across a range of test parameters. In experiment I, dichotic and monaural FM detection was measured as a function of duration and modulator starting phase. Dichotic FM thresholds were lower than monaural FM thresholds and the modulator starting phase had no effect on detection. Experiment II measured monaural FM detection for signals that differed in modulation rate and duration such that the improvement with duration in seconds (carrier) or cycles (modulator) was compared. Monaural FM detection improved monotonically with the number of modulation cycles, suggesting that the modulator is extracted prior to detection. Experiment III measured dichotic FM detection for shorter signal durations to test the hypothesis that dichotic FM relies primarily on the signal onset. The rate of improvement decreased as duration increased, which is consistent with the use of primarily onset cues for the detection of dichotic FM. These results establish that improvement with duration occurs as a function of the modulation cycles at a rate consistent with the independent-samples model for monaural FM, but later cycles contribute less to detection in dichotic FM.

## I. INTRODUCTION

The auditory perception of the instantaneous amplitude or temporal fine structure (TFS) of sound is important for speech communication (e.g., Strelcyk and Dau, 2009; Johannesen *et al.*, 2016) but susceptible to impairment related to aging, hearing loss, and traumatic injury (e.g., Lacher-Fougère and Demany, 2005; Gallun *et al.*, 2013; Füllgrabe *et al.*, 2015; Hoover *et al.*, 2017). The threshold of detection of frequency modulation (FM) has been used as a sensitive and efficient index of TFS (Hoover *et al.*, 2019). The role of TFS in the detection of monaural FM remains a continued area of debate (Verschooten *et al.*, 2018), but both monaural and dichotic FM detection worsen with hearing loss and increasing age (Lacher-Fougère and Demany, 1998; Moore and Skrodzka, 2002; He *et al.*, 2007; Grose and Mamo, 2012; Wallaert *et al.*, 2016; Whiteford *et al.*, 2020), and FM detection has been shown to predict individual differences in the benefit of hearing aids (Lopez-Poveda *et al.*, 2017). The present study evaluated the improvement in monaural and dichotic FM detection as a function of increasing duration. We hypothesized that the detection of monaural FM would reflect optimal integration of the signal throughout its duration, consistent with the independent-samples model. Dichotic FM contains an additional interaural time difference (ITD) cue that has been shown to result in detection at a lower depth compared to monaural. The use of this cue was predicted to result in dichotic FM detection relying more on cues toward the onset of the signal, resulting in an improvement with increasing duration that would be consistent with the independent-samples model only at short durations and gradually level off.

Although it has been shown that FM detection is dependent on certain stimulus characteristics, such as modulation frequency (Green *et al.*, 1976; Hartmann and Klein, 1980; Moore and Sęk, 1995; Moore and Skrodzka, 2002; Ernst and Moore, 2012; Whiteford and Oxenham, 2015; Wallaert *et al.*, 2016; Wallaert *et al.*, 2018), carrier frequency (Moore and Sęk, 1995; Moore and Skrodzka, 2002; He *et al.*, 2007; Ernst and Moore, 2012), and the combination of the duration and number of modulation cycles (Hartmann and Klein, 1980; Grose and Mamo, 2012; Wallaert *et al.*, 2016; Wallaert *et al.*, 2018; Hoover *et al.*, 2019; Koerner *et al.*, 2020), there are still many questions remaining regarding the stimulus parameters that impact FM detection and the physiological cues by which dynamic changes in frequency are encoded by the auditory system.

For low modulation frequencies (i.e., FM < 10 Hz; Moore and Sęk, 1995), it is assumed that the detection of FM is based on a temporal coding mechanism. Either the instantaneous amplitude of the signal is encoded by phase-locked firing (i.e., TFS of the stimulus) or FM is converted to amplitude modulation (AM) by changes in the output of the auditory filters fixed in place along the cochlear partition (Zwicker, 1956; Khanna and Teich, 1989; Sęk and Moore, 1995; Moore and Sęk, 1996). Differences in the relationship between the modulation frequency and detection for FM and AM signals have previously been interpreted as evidence for the importance of TFS cues for FM detection at low modulation frequencies (Rose *et al.*, 1967; Moore and Sęk, 1995; Parthasarathy *et al.*, 2019). However, there is converging evidence that place cues alone may be able to explain FM detection even at low modulation rates. A model of the representation of place in the auditory cortex shows that the low thresholds observed for FM at low modulation frequencies can be obtained by combining the output of multiple critical bands (Micheyl *et al.*, 2013). Behavioral evidence includes highly correlated AM and FM detection thresholds within individuals (Whiteford and Oxenham, 2015; Paraouty and Lorenzi, 2017), as well as comparable thresholds for FM detection and an AM detection task in which out-of-phase modulators were presented at neighboring carrier frequencies to simulate the excitation pattern of an FM signal (Whiteford *et al.*, 2020).

Physiological and computational models of FM detection must be able to account for the differences in the rate of improvement between monaural and dichotic FM as well as changes in the rate of improvement with duration. For monaural FM, the most relevant work on the impact of stimulus duration on FM detection is that of Hartmann and Klein (1980), who performed an experiment to determine the improvement in FM detection with increasing signal duration measured in the number of modulation cycles presented to young listeners with normal hearing. Using stimuli with a low carrier frequency of 800 Hz and low modulation frequency of 4 Hz, the number of modulation cycles presented was systematically varied, but overall stimulus duration covaried with the number of modulation cycles. As the number of modulation cycles was increased from one to four, FM detection improved markedly. Similar results were reported by Wallaert *et al.* (2018). However, because the number of cycles covaried with overall signal duration, it is unclear whether the observed improvement in FM detection threshold was due to the increased number of modulation cycles presented (from 1 to 4) or the resulting increase in stimulus duration (from 250 to 1000 ms). If the improvement in FM detection is best described as a function of the number of cycles, independent of the total stimulus duration, this result would be similar to the observed improvement with cycles in AM detection (Viemeister, 1979; Lee, 1994) and could provide evidence for a similar detection mechanism underlying the coding of AM and FM stimuli. The temporal-envelope model, implemented by Wallaert *et al.* (2018), failed to account for low-rate FM thresholds in young normal-hearing listeners. One interpretation of this failure is that different mechanisms are responsible for low-rate AM and FM detection, where the latter is presumed to rely on TFS cues. It remains unclear whether low-rate FM detection improves with the number of cycles of the modulator, as in AM detection, or with overall signal duration independent of the number of cycles of the modulator.

An additional detection cue is associated with dichotic FM, created by presenting an FM tone of a given rate to one ear and a static tone to the other ear (Green *et al.*, 1976; Witton *et al.*, 2000) or by presenting FM in which the modulation phase differs between the ears (Grose and Mamo, 2012; Whiteford and Oxenham, 2015). The interaural difference gives rise to a binaural cue that is not present in monaural FM and results in substantially lower detection thresholds (Green *et al.*, 1976; Witton *et al.*, 2000). This binaural FM cue is thought to rely on differences in the time of arrival of auditory neuron action potentials at binaural comparator units in the brainstem (Moushegian *et al.*, 1967; Goldberg and Brown, 1969; Caird and Klinke, 1983; Yin and Chan, 1990; Stecker and Gallun, 2012). For this cue to be extracted by the binaural system, it is essential that the interaural difference is precisely reflected in the phase-locked patterns of the auditory-nerve responses. Because dichotic FM detection thresholds are much lower (better) than diotic FM detection thresholds, dichotic FM detection is thought to reflect the use of an interaural difference cue rather than the addition of another monaural cue from the second ear (Grose and Mamo, 2012).

Precise timing may reflect the TFS of the carrier signal or, in the case of FM-to-AM conversion, the binaural system is assumed to compute differences in the relative arrival time of fluctuations in the amplitude envelope. Both fine structure and envelope differences can be expressed as an ITD. In dichotic FM, the ITD is sinusoidal with a frequency related to the frequency of the modulator (in the case of the stimuli used in the experiments below, 2 Hz), and an amplitude (magnitude of ITD) that is related to the modulation depth and carrier frequency.

In monaural FM detection, temporal cues are assumed to be weighted equally throughout the duration of the stimulus, resulting in better detection for longer signals. This is not necessarily the case for binaural tasks like dichotic FM in which the relative contribution of onset and ongoing ITD cues can result in suboptimal improvement with duration (Stecker and Gallun, 2012). To our knowledge, the potential unequal temporal weighting of onset compared to the ongoing ITD has not been studied for dichotic FM. Stimulus characteristics, such as bandwidth and spectral density, influence the extent to which detection relies on the onset (Freyman *et al.*, 1997), and dichotic FM represents a combination of stimulus characteristics for which the extent of onset dominance remains unclear. The effects of duration on binaural sensitivity measured with fluctuating ITD have been investigated in previous studies (e.g., McFadden and Moffitt, 1977; Hafter *et al.*, 1979; Stecker and Hafter, 2002). These studies showed that ITD is detected and localized with an onset-dominated temporal weighting function, where cues from the onset are weighted more heavily than cues extracted from the ongoing portion of the stimulus regardless of whether this weighting results in optimal detection (Hafter *et al.*, 1979; Stecker and Hafter, 2002; Stecker and Bibee, 2014; Dietz *et al.*, 2014). Dichotic FM stimuli are sparse and tonal but vary in frequency over some bandwidth. Sparse tonal signals and narrowband noise signals both demonstrate strong onset dominance (Freyman *et al.*, 1997; Freyman and Zurek, 2017), suggesting that dichotic FM detection should be dominated by onset cues. The current investigation systematically compared effects of duration on monaural and dichotic sensitivity using matched stimuli in the same participants. If onset ITD dominates the detection of dichotic FM, we would expect a pattern of improvement with duration that differs from monaural FM.

Three experiments were performed with the goal of evaluating FM across a range of test parameters used in previous studies that support the use of FM detection in the clinic. Specifically, this study examined duration and modulation phase effects that differed across previous studies. The results provide practical guidance on the design of test protocols and interpretation of results across studies that use different methods. The primary goal of experiment I was to compare the rate of improvement in FM detection thresholds with increasing duration between monaural and dichotic stimulus conditions. A difference in the starting phase of the modulator, resulting from different equations used to generate stimuli across studies, was also evaluated. The goal of experiment II was to determine whether monaural FM detection for low modulation frequencies depends on the stimulus duration, the number of modulation cycles, or some combination of the two. Experiment III tested a wider range of stimulus durations than Experiment I to evaluate the hypothesis that dichotic FM detection relies primarily on the signal onset consistent with similar ITD tasks. If dichotic FM detection primarily relies on onset cues, the rate of improvement for dichotic FM, unlike monaural FM, will change with duration. The results were interpreted in the context of the independent-samples model of improvement with increasing signal duration as well as competing models of the role of TFS cues in monaural and dichotic FM detection.

## II. EXPERIMENT I: EFFECTS OF STIMULUS DURATION AND MODULATOR PHASE ON MONAURAL AND DICHOTIC FM STIMULI

### A. Background

Two different equations have been used to specify FM signals in psychoacoustics research, and the equations differ in the starting phase of the modulator. The equation for sinusoidal FM, which is typically used, is

$$x\left(t\right)=\text{sin}\;\left[2\pi f_{c}t+\beta \;\;\text{sin}\;\left(2\pi f_{m}t\right)\right],$$ (1)

where $f_{c}$ is the carrier frequency, $f_{m}$ is the modulation frequency, and β is the modulation index. The midline-to-peak modulation depth, $f_{d}$, is related to the modulation index by $f_{d}=\beta /2f_{m}$ (e.g., Witton *et al.*, 2000; Grose and Mamo, 2012). The sin function inside the brackets serves as a phase term, adding or subtracting to the accumulated phase of the carrier over time. In Eq. (1), the modulator starts in “negative cosine” (-cos) phase, which means that in the dichotic condition, the instantaneous frequencies at the left and right ears are identical at time zero. However, this corresponds to the lowest frequency of the modulated signal at the left ear and the highest frequency of the modulated signal at the right ear. To start the modulator in sin phase, such that the frequency at each ear at time zero corresponds to the center frequency of the modulation at each ear, an additional phase term must be added to the modulator, which simplifies to

$$x\left(t\right)=\text{sin}\;\left[2\pi f_{c}t+\beta \left(1-\text{cos}\;\;2\pi f_{m}t\right)\right].$$ (2)

**FIG. 1.**
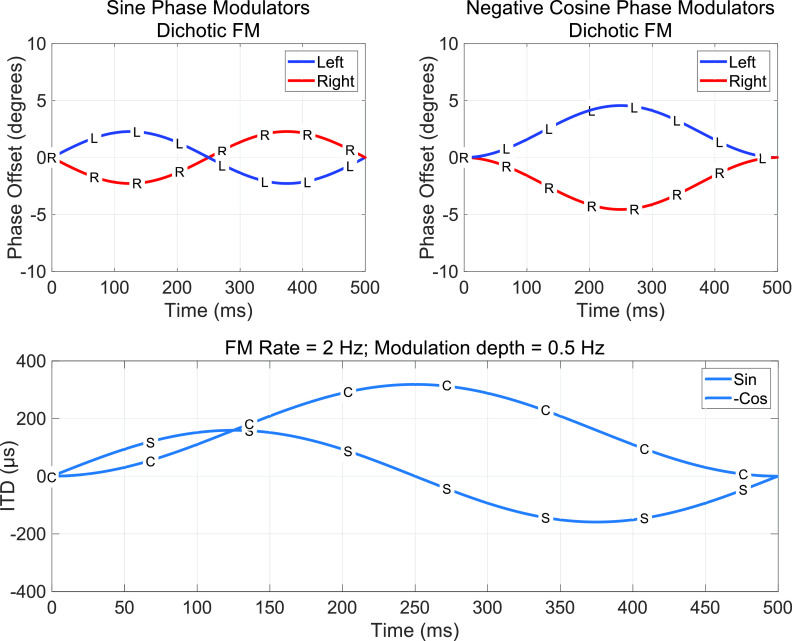
(Color online) Analysis of dichotic FM via a comparison of the signals at the two ears. (A) (sin) and (B) (-cos) illustrate phase offset as a function of time throughout the stimulus duration. (C) illustrates the instantaneous ITD throughout the duration of the stimulus for the sin (*S*) and -cos (*C*) stimuli.

The signals defined by Eqs. (1) and (2) are illustrated in Fig. 1, where the phase of the signals at each ear are shown as a function of time in Figs. 1(A) and 1(B) and the resulting ITDs are shown in Fig. 1(C). The important point is that the ITD for the sin phase contains two maxima (one positive at 125 ms and one negative at ∼375 ms) during each cycle, and these ITDs are centered around the midline. The negative cosine phase, on the other hand, has a single maximum ITD that occurs in the middle of the cycle (250 ms) and a value that corresponds to twice the maximum positive ITD value for the sin phase stimulus. If the two signals are compared in terms of the maximum positive ITD, then the -cos signal appears to have a greater maximum ITD. But, if the peak-to-peak difference is considered, then the two methods produce identical maximum ITD values.

Comparing performance with these two starting phase terms as a function of duration has significant value for potentially helping to understand the mechanism of the interaural phase difference (IPD)/ITD extraction for dichotic FM stimuli. For example, if detection is based on the maximum instantaneous ITD and the stimulus duration is 500 ms or greater, then thresholds should be better for the -cos condition. If, on the other hand, detection is based on the maximum peak-to-peak difference, then there should be no difference between sin and -cos. This also has implications for the interactions between ITD and duration, although the specific predictions depend not only on changes in the instantaneous ITD over time but also the temporal weighting functions, which have never been studied for FM stimuli.

In the present study, the influence of starting phase of the modulator was addressed in both monaural and dichotic conditions by using two forms of modulation, FM detection with a -cos starting phase, as in Eq. (1), and FM with a sin starting phase, as in Eq. (2). In the monaural case, there was no reason to expect that the starting phase of the modulator should have any effect on the detection thresholds over the range of durations tested. For the dichotic stimuli, however, the duration of the stimulus could potentially interact with ITD sensitivities in the manner discussed above. The first question to be answered was whether or not the starting phase has any effect on dichotic FM detection. If not, there is less need to explore the exact predictions of the interactions with duration.

It has been shown that monaural FM detection improves with increasing duration (Hartmann and Klein, 1980); however, the effect of duration has not been evaluated for dichotic FM detection. To compare the rate of improvement with increasing duration across monaural and dichotic conditions, FM detection thresholds were measured as a function of stimulus duration at a fixed modulation frequency of 2 Hz.

Three hypotheses were evaluated in experiment I. The first was that, consistent with previous studies, dichotic FM detection thresholds would be lower (better) than monaural thresholds due to the availability of ITD cues in the dichotic condition. The second hypothesis was that monaural FM detection would improve with increasing signal duration, but dichotic FM detection would show minimal improvement over this range of duration. Less improvement with duration in the dichotic condition would provide evidence of reliance on primarily onset cues, which is consistent with similar tasks relying on ITD. This is in contrast to monaural FM detection for which improvement with duration occurs steadily over a wide range of duration (Hartmann and Klein, 1980; Wallaert *et al.*, 2018). Finally, the third hypothesis was that dichotic FM thresholds would be lower in the -cos modulation starting phase condition compared to the sin condition, which is consistent with the use of maximum instantaneous ITD cues for detection.

### B. Participants

Participants included ten adults between 20 and 24 years of age (eight females) with audiometrically normal hearing [≤20 dB hearing level (HL) at octave frequencies between 250 and 8000 Hz; ANSI, 2010]. All participants provided written consent for study participation, and all procedures were approved by the university Institutional Review Board. Data collection was completed over the course of 4–6 sessions, lasting approximately 2 h each, with a total of 8–12 h of testing per participant. Participants were compensated at an hourly rate.

### C. Stimuli

A total of 16 conditions were tested, including a combination of 4 stimulus durations (1250, 1000, 750, and 500 ms), 2 modulation starting phases (sin and -cos), and either monaural (right ear only) or dichotic presentation. In the dichotic condition, FM stimuli were presented to both ears with the modulator phase inverted at one ear, which is consistent with the FM/FM condition from Grose and Mamo (2012). To minimize the presence of a cue related to the frequency of the stimulus measured at a single point in time, the carrier frequency was randomly (uniform distribution) selected in each interval between 460 and 540 Hz with a 1-Hz resolution. This encouraged listeners to actively track the change in frequency over time rather than simply choosing the interval with a frequency that differed from the standard, unmodulated tone. The same carrier frequency was used in both ears in the dichotic condition but note that stimuli generated with Eq. (1) started at a different instantaneous frequency due to the modulator starting phase. The sin or -cos modulator starting phase was chosen so that in both starting phase conditions, the frequency initially increased in the monaural condition or initially increased in the right ear and decreased in the left (rightward motion) in the dichotic condition (e.g., Fig. 1). Thus, the absolute range of frequencies traversed over the course of a cycle of modulation was equal in both the sin and -cos starting phase conditions. The standard interval consisted of an unmodulated pure tone, whereas the signal interval consisted of a pure tone with a 2-Hz modulation frequency. All stimuli were shaped with a 20-ms cosine-squared rise/fall window. The presentation level was 65 dB sound pressure level (SPL). Stimuli were generated in matlab (MathWorks, Inc., 2000) and delivered via a USB soundcard (ESI, U24XL, Baden-Württemberg, Germany) to circumaural headphones (Sennheiser, HD 280 Pro, Wedemark, Germany).

### D. Procedures

Stimuli were presented in a four-interval, two-alternative forced choice method, following the methods of Hoover *et al.* (2019), in which the first and last intervals always consisted of the unmodulated standard stimulus. The second and third intervals consisted of either the standard stimulus or modulated target stimulus with an equal probability of the target stimulus occurring in either interval two or interval three during each trial. This approach reduced the influence of memory or attention on performance as the comparison to the standard can always be made either forward or backward in time across intervals, unlike in a two- or three-interval task. This is important because Gallun *et al.* (2012) showed that comparisons to preceding standards yield better performance than comparisons to standards that follow the target. Visual feedback was presented after each trial to indicate a correct or incorrect response. The participant was seated comfortably in front of a computer screen and testing was conducted in a sound attenuating chamber. The graphic user interface generated in matlab consisted of four buttons in a horizontal row across the screen corresponding to the respective four intervals. As the stimulus in each interval was presented, the corresponding button was illuminated. Participants were instructed to choose the button corresponding to the interval containing the stimulus that they perceived to be “different” with a click of the computer mouse. FM detection thresholds were estimated using a three-down, one-up adaptive tracking procedure (Levitt, 1971). The initial step size was a factor of 2, doubling or halving the modulation depth in Hz. After the first two reversals, a smaller step size of 2^1/4^ was used for the remaining eight reversals. A threshold estimate for each test run was calculated based on the geometric mean of the last six reversals. The final threshold estimate for a condition was calculated from the geometric mean of the three test run thresholds. For each condition, one practice run was performed, followed by three test runs. Duration, starting phase, and monaural or dichotic conditions were tested in random order for each participant.

### E. Statistical analyses

The data were analyzed using a repeated-measures analysis of variance (ANOVA) to evaluate three predictions: (1) monaural FM thresholds will be significantly higher than dichotic FM thresholds, as indicated by a significant main effect of condition; (2) the rate of improvement will be significantly different between the monaural and dichotic conditions, as indicated by a significant interaction between condition and duration; and (3) modulation starting phase will have a significant effect on thresholds in the dichotic but not monaural condition, as indicated by a significant interaction between starting phase and condition.

### F. Results

**FIG. 2.**
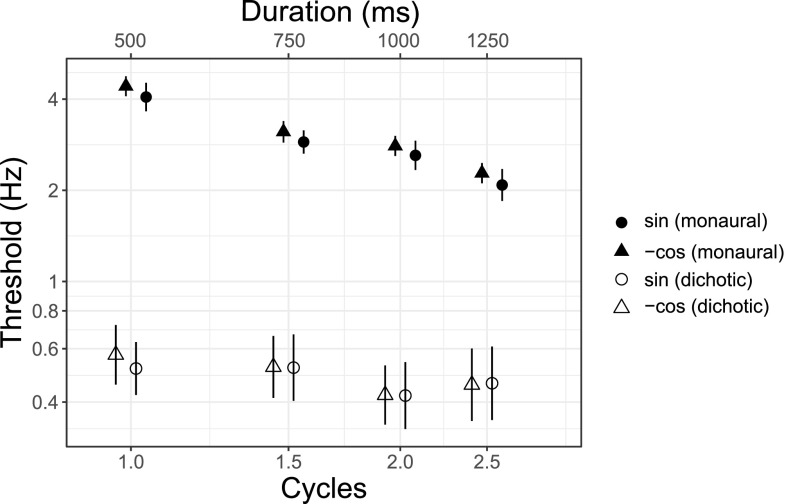
Experiment I: The effect of stimulus duration on monaural and dichotic FM detection. Mean FM detection thresholds are shown with standard error bars for monaural (closed symbols) and dichotic (open symbols) conditions with the modulation starting phase indicated by the symbol as noted in the key.

Figure 2 shows mean FM detection thresholds as a function of the number of modulation cycles. Thresholds for the monaural condition (closed symbols) decreased as a function of duration from 1 to 2.5 cycles (500–1250 ms). Thresholds for the dichotic condition (open symbols) were lower than those for the monaural condition. The distribution of both of monaural and dichotic thresholds were consistent with previous threshold estimates for the detection of 2 Hz dichotic FM for a four-cycle (1250-ms) stimulus duration (Grose and Mamo, 2012; Hoover *et al.*, 2019).

FM detection thresholds were analyzed using a three-way repeated-measures ANOVA with fixed effects of condition (two levels, monaural and dichotic), duration (four levels), and starting phase (two levels, sin and -cos), all two- and three-way interactions, and a random effect of participant (ten levels). The analyses were performed on the log transform of the data. The main effect of condition was statistically significant (*F*_1,135_ = 807.586, *p* < 0.001) as was duration (*F*_3,135_ = 8.577, *p* < 0.001). The starting phase (*F*_1,135_ = 0.695, *p* = 0.41) was not significant. The three-way interaction (*F*_3,135_ = 0.044, *p* = 0.99), condition by duration (*F*_3,135_ = 2.65, *p* = 0.051), condition by phase (*F*_1,135_ = 0.184, *p* = 0.67), and duration by phase (*F*_3,135_ = 0.045, *p* = 0.99) were not significant. Multiple paired *t*-tests with Bonferroni correction were performed *post hoc* to evaluate differences among the four signal durations in the monaural and dichotic conditions separately. Significant differences were found between monaural threshold means at each duration compared to every other duration at the *p* < 0.05 level with the exception of the mean thresholds at 750 and 1000 ms (*p* = 0.25). A significant difference was found between dichotic threshold means at 500 and 1000 ms (*p* = 0.046), but no other significant differences were found between dichotic threshold means among the four durations at the *p* < 0.05 level.

### G. Discussion

Consistent with previous studies that tested FM detection only for 2.5 cycles at 2 Hz (1250 ms; Grose and Mamo, 2012; Hoover *et al.*, 2019), thresholds in the dichotic condition were lower (better) than those in the monaural condition, consistent with the use of a fundamentally different cue for detection in monaural and dichotic FM conditions. The geometric mean and variance of thresholds at 2.5 cycles were comparable to data from those previous studies. There was a main effect of duration, consistent with an improvement in FM detection with increasing duration (Hartmann and Klein, 1980; Wallaert *et al*, 2018), but there was no interaction between the monaural and dichotic conditions, suggesting that there was no difference in improvement with duration between the conditions. This result was surprising given our *a priori* hypothesis that the use of an onset ITD cue to detect dichotic FM would limit improvement with duration. There was no evidence of a difference in the modulation starting phase in either monaural or dichotic detection at any duration. The lack of a difference in detection thresholds with starting phase in the dichotic conditions suggests that listeners rely on peak-to-peak and not maximum ITD.

## III. EXPERIMENT II: CARRIER AND MODULATOR EFFECTS IN MONAURAL FM DETECTION

### A. Background

Hartmann and Klein (1980) used the standard signal-detection theoretic approach (Green and Swets, 1974) of modeling the decrease in the detection thresholds with increasing duration whereby each unit of time results in an independent sample of the information needed to do the task. These multiple independent samples are assumed to be drawn from a normally distributed information source (a hypothetical neural information extraction process) that has a mean that corresponds to the modulation frequency and a variance that is dependent on the neural process. This class of independent-samples model predicts that the variance of the estimate of the mean value of the neural process will decrease as a function of the number of looks and, thus, performance (if measured in a manner that depends on the standard deviation of the estimate) will improve with increasing duration at a rate of $n^{-1/2}$, where *n* is the number of looks. The predictions of such a model, at least for the monaural case, are expressed in Eq. (3).

Independent-samples duration model,

$$f_{d}\left(t\right)=f_{d}\left(t_{\text{reference}}\right)/\sqrt{t},$$ (3)

where $f_{d}$ is the modulation depth in Hz at duration (in seconds) t, $f_{d}\left(t_{\text{reference}}\right)$ is the FM detection threshold (Hz) at a reference point such as 500 ms (one cycle). Similarly, one can formulate a model that is a function of duration as measured in the number of modulation cycles in the stimulus.

Independent-samples cycles model

$$f_{d}\left(n\right)=f_{d}\left(n_{\text{reference}}\right)/\sqrt{n},$$ (4)

where threshold in the numerator is defined as above, and n is the duration in cycles for both the reference threshold and the threshold to be predicted. The independent-samples model, in both seconds and cycles, is typically specified as shown in Eqs. (3) and (4) with $f_{m}$ and duration specified in linear units. However, FM detection thresholds are typically reported on a log scale (e.g., Whiteford and Oxenham, 2015; Wallaert *et al.*, 2016). If we transform the independent-samples model equation by taking the log of both sides, the result is a convenient equation,

$$\text{log}\;\left[f_{d}\left(d\right)\right]=\text{log}\;\left[f_{d}\left(d_{r}\right)\right]-0.5d,$$ (5)

where d is the duration in either seconds [Eq. (3)] or cycles [Eq. (4)], $d_{r}$ is the reference duration, and $f_{m}\left(d_{r}\right)$ is the FM detection threshold at the reference duration in Hz. A commonly used logarithmic base for Hz is two, which gives a result in octaves relative to some reference frequency. Equation (5) shows that the independent-samples model predicts an improvement in FM detection that is a straight line with a slope of -0.5 octaves per doubling of the duration.

Several studies have shown that improvement in AM detection is best described as a function of the number of cycles, independent of total stimulus duration (e.g., Viemeister, 1979; Lee, 1994; Lee and Bacon, 1997). It is unclear whether an improvement in FM detection can be similarly predicted from modulation cycles or if thresholds are better described as a function of stimulus duration. The distinction between these cues could have implications for explaining temporal coding mechanisms of low-rate (<10 Hz), low carrier FM. Many believe that FM is detected using phase-locked firing of the auditory nerve to the fine structure of the carrier (see Moore, 2020, for a recent review). In this case, we expect a signal with a longer overall duration to yield an improvement in detection independent of the number of modulation cycles because more cycles of the carrier are available for the extraction of a phase-locking cue. An improvement in FM detection as a function of the duration would provide evidence that TFS cues are being encoded directly by the monaural system. Alternatively, TFS cues could be converted to AM at the periphery as described by the FM-to-AM conversion hypothesis (Zwicker, 1956; Khanna and Teich, 1989). In this case, an improvement in detection should be best described as a function of the total cycles of the modulator, which is consistent with the AM studies mentioned above.

To explore the possible effects of stimulus duration in seconds and number of modulation cycles on monaural FM detection, a wider range of cycles than were included in experiment I was evaluated. Parametric manipulation of duration and number of cycles required that the modulation frequency be varied accordingly (0.5–8 Hz; see Table I). Three possible outcomes were anticipated. If FM detection depends on the overall stimulus duration in seconds, then thresholds should be lower for the 1250-ms than for the 500-ms stimulus durations and not vary with the number of cycles. If FM detection depends on the number of modulation cycles, then thresholds should decrease with the number of cycles and not vary with duration in seconds. Finally, if FM depends on both duration in seconds and number of cycles, then there should be parallel improvement with increasing cycles with the 1250-ms stimuli leading to lower overall thresholds than the 500-ms stimuli.

**TABLE I.** Matrix of stimulus conditions based on duration, modulation frequency, and number of cycles.

	Modulation frequency (Hz)				
Duration (ms)	0.5	1	2	4	8
500	0.25 cycles	0.5 cycles	1 cycle	2 cycles	4 cycles
1250	0.625 cycles	1.25 cycles	2.5 cycles	5 cycles	—

**FIG. 3.**
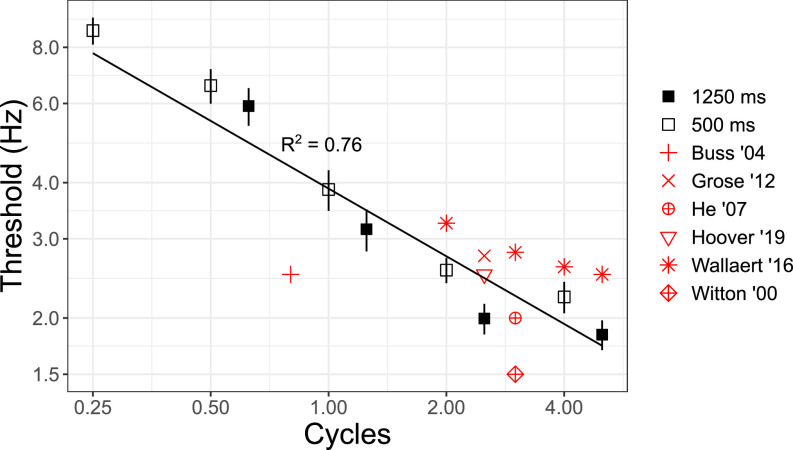
(Color online) Experiment II: The effect of the number of modulation cycles on monaural FM detection. Mean monaural FM detection thresholds are shown with standard error bars for stimulus durations of 500 ms (open black symbols) and 1250 ms (closed black symbols) as a function of modulation cycles. The black line represents predictions made by the independent-samples cycles model of Eq. (4) with the corresponding adjusted *R*^2^ value. Data from other studies as indicated in the legend include Buss *et al.*, 2004; Grose and Mamo, 2012; He *et al.*, 2007; Hoover *et al.*, 2019; Strelcyk and Dau, 2009; Wallaert *et al.*, 2016; and Witton *et al.*, 2000. See Table II for details of the modulation and carrier frequencies used in those studies.

**TABLE II.** Monaural FM detection thresholds from studies using modulation rates and carrier frequencies that are similar to those in the present study. Thresholds were included in Fig. 3 at the appropriate duration in cycles.

Study	*f_m_* (Hz)	*f_c_* (Hz)	Duration (ms)	Duration (cycles)	Threshold (Hz)
Buss *et al.*, 2004	2	500	400	0.8	2.5
He *et al.*, 2007	5	500	1500	3	2
Strelcyk and Dau, 2009	2	750	750	1	2.75
Witton *et al.*, 2000	2	500	1500	3	1.5
Hoover *et al.*, 2019	2	500	1250	2.5	2.5
Grose and Mamo, 2012	2	500	1250	2.5	2.75
Wallaert *et al.*, 2016	2	500	1000	2	3.25
Wallaert *et al.*, 2016	2	500	1500	3	2.8
Wallaert *et al.*, 2016	2	500	2000	4	2.6
Wallaert *et al.*, 2016	2	500	2500	5	2.5

### B. Participants

The threshold for detecting FM was measured in ten participants between the ages of 20 and 24 years old (all female; seven returned from experiment I) with audiometrically normal hearing (≤20 dB HL at octave frequencies between 250 and 8000 Hz). All participants provided written consent for study participation, and all procedures were approved by the university Institutional Review Board. Data collection was completed over the course of 4–6 sessions, lasting approximately 2 h each, with a total of 8–12 h of testing per participant. Participants were compensated at an hourly rate.

### C. Stimuli

To establish the stimulus parameters for this experiment, we considered five possible modulation frequencies (0.5, 1, 2, 4, 8 Hz) and two stimulus durations (500 or 1250 ms) such that the combination of the modulation frequency and number of cycles resulted in different numbers of modulation cycles (Table I). Thus, a total of nine conditions were tested. All stimuli were presented monaurally to the right ear and with a sin starting phase. The carrier frequency rove and presentation level were identical to those in experiment I as were all other aspects of the signal generation and presentation.

### D. Procedures

All aspects of the procedure were identical to those in experiment I. Duration and modulation frequency were tested in random order for each participant.

### E. Statistical analyses

The data were analyzed to test for a statistically significant deviation from the improvement in threshold with increasing duration predicted by the independent-samples model. Hierarchical regression was performed to examine whether the number of modulation cycles presented explains a statistically significant amount of variance in performance after accounting for the effects of stimulus duration.

### F. Results

Figure 3 shows mean FM detection thresholds with standard error bars as a function of the number of cycles in the stimulus for durations of 500 ms (open black symbols) and 1250 ms (closed black symbols). Modulation detection thresholds decreased monotonically with increasing number of cycles. If improvement occurred as a function of time in seconds, then there would be no difference between the stimuli with the same duration (i.e., open symbols would each have the same threshold; closed symbols would each have the same threshold). Clearly, this was not the result.

**FIG. 4.**
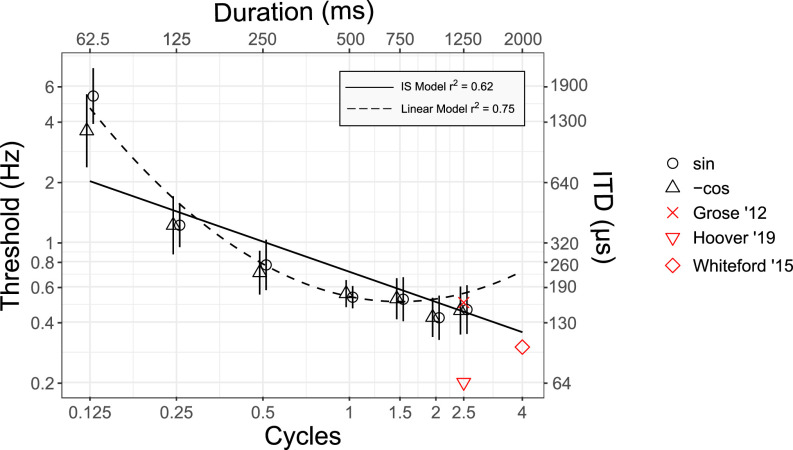
(Color online) Experiment III: Effect of short stimulus duration on dichotic FM detection. Mean dichotic FM detection thresholds are shown with standard error bars as a function of modulation cycles. Modulation starting phase is indicated by the symbol as shown in the key. The functions represent predictions of different model fits to the data with corresponding adjusted *R*^2^ values shown in the inset. Data from other studies include Grose and Mamo, 2012; Whiteford and Oxenham, 2015; and Hoover *et al.*, 2019. See Table IV for details about the modulation rate and duration.

To examine the unique contribution of modulation cycles in predicting the monaural FM detection performance, a hierarchical multiple regression analysis was performed. Variables that explain FM detection threshold were entered in two steps. In step 1, threshold in log_2_(Hz) was the dependent variable and duration was the independent variable. The results of step 1 indicated that the variance accounted for (*R*^2^) with the single independent variable of duration equaled 10.2% (*R*^2^ = 0.102), which was statistically significant (*F*_1,88_ = 9.944, *p* < 0.01). In step 2, an additional independent variable, cycles, was entered into the regression equation. The addition of cycles to the regression model explained 49.7% of the variation in the threshold (Δ*R*^2^ = 0.497), and this change in *R*^2^ was statistically significant (*F*_2,87_ = 64.914, *p* < 0.01). When both duration and cycles were included in step 2 of the regression model, both independent variables were significant predictors of the threshold and, together, account for 59.9% of the variance in threshold. However, the most important predictor of threshold was cycles, which uniquely explained 49.7% of the variation in threshold. Regression statistics are reported in Table III.

**TABLE III.** The summary of the hierarchical regression analysis for variables predicting monaural FM detection threshold. Note *p < 0.1; **p < 0.05; ***p < 0.01.

Variable	β	*t*	sr^2^	*R*^2^	Δ*R*^2^
Step 1				0.102	0.102
Duration	−0.001***	−3.153	0.102		
Step 2				0.599	0.497
Duration	−0.0003*	−1.916	0.017		
Cycles	−0.398***	−10.383	0.497		

**TABLE IV.** Stimulus parameters and dichotic FM detection thresholds from recent studies that used stimulus parameters similar to this experiment. Thresholds are plotted in Fig. 4 as single (red) symbols for comparison with the present study.

Study	*f_m_* (Hz)	*f_c_* (Hz)	Duration (ms)	Duration (cycles)	Threshold (Hz)
Whiteford and Oxenham, 2015	1	500	2000	4	0.3
Grose and Mamo, 2012	2	500	1250	2.5	0.5
Hoover *et al.*, 2019	2	500	1250	2.5	0.2

The intercept of the line fit to the data with a fixed slope of −0.5 octaves per doubling of duration in cycles at 1 cycle was 1.94 log_2_(Hz), 95% confidence interval (CI) 1.75, 2.12 log_2_(Hz), root-mean-square error (RMSE) 0.428 log_2_(Hz). The model line and adjusted *R*^2^ value were plotted in Fig. 3. Data for the 500 ms and 1250 ms durations appeared to be well-fit by the independent-samples model as a function of the number of cycles.

### G. Discussion

The results are consistent with the independent-samples model as a function of cycles [Eq. (4)] rather than stimulus duration in seconds [Eq. (3)]. Note that the significant effect of duration in seconds observed in the hierarchical regression represents the fact that duration in seconds covaried with the number of cycles when combining across the modulation rates. Because different modulation frequencies were used to vary the number of cycles presented in a given duration, duration in seconds was independently manipulated only within each modulation rate. Had duration in seconds influenced detection, we would expect Fig. 3 to show thresholds for the 1250 ms duration (filled squares) to be lower than thresholds for the 500 ms duration. This was not the case; all thresholds were on the same line with a slope of -0.5 octaves per doubling of the number of cycles. If one assumes that FM detection is based on the same underlying process across the evaluated modulation frequencies, then it appears that over the range of conditions evaluated in experiments I and II, monaural FM detection depends on the number of modulation cycles and is independent of overall duration. This improvement with cycles of the modulator is consistent with that seen in AM detection tasks.

## IV. EXPERIMENT III: ONSET DOMINANCE IN DICHOTIC FM

### A. Background

Improvement in monaural FM detection with increasing duration is consistent with the independent-samples cycles model [Eq. (4)] as shown in experiment II. If dichotic FM is like other tonal ITD tasks, then we should expect improvement with increasing duration to have a shallower slope than predicted by the independent-samples model, reflecting suboptimal use of samples arriving later in the signal (e.g., Stecker and Bibee, 2014). This pattern of improvement has been shown using similar stimuli in ITD tasks in which a narrowband signal was used and there was no clear onset cue to ITD (Ricard and Hafter, 1973; Hafter *et al.*, 1979; Freyman and Zurek, 2017). However, no significant interaction was observed in experiment I between duration and condition, providing no evidence that improvement with duration differed between monaural and dichotic FM conditions. This may have been due to variability in threshold estimates in dichotic FM. In experiment I, the difference between the shortest (500 ms) and longest (1250 ms) stimuli was 2.5 times. Over that range, given the low thresholds for dichotic FM detection (overall mean 1.5 Hz), the independent-samples model predicts an improvement of only 0.2 Hz. Given the magnitude of the anticipated effect, we were unable to reliably test the hypothesis that the rate of improvement in dichotic FM thresholds with increasing duration was less than that predicted by the independent-samples model using data collected in experiment I.

To examine a range over which the independent-samples model predicts larger changes in threshold for dichotic FM detection, data from experiment I were combined with a set of shorter durations tested in experiment III. If this task relies on the onset, similar to other ITD tasks, we should see improvement with duration consistent with the independent-samples model up to about 300 ms (0.6 cycles at 2 Hz), and then gradual deviation from the independent-samples slope as duration increases (Ricard and Hafter, 1973; Hafter *et al.*, 1979). Experiment III was designed to test for a slowing in the rate of improvement with increasing signal duration above 300 ms, which we would expect due to the use of ITD cues favoring the onset.

FM depth can be transformed into ITD using the equations provided by Witton *et al.* (2000), which can be combined to show the relationships between the relevant signal parameters,

$${\text{ITD}}_{\text{peak}-\text{to}-\text{peak}}=\frac{2f_{d}}{\pi f_{c}f_{m}}.$$ (6)

Here, peak-to-peak ITD is shown to depend on $f_{d}$, which is the modulation depth (in Hz), $f_{c}$, which is the carrier frequency in Hz, and $f_{m}$, which is the modulation rate in Hz. Peak-to-peak ITD is the total deviation in ITD from the left to right during the stimulus as shown in Fig. 1. In experiment I, no difference was found between sin and -cos modulation starting phases, which is consistent with the use of peak-to-peak modulation depth. However, if ITD is calculated using peak-to-peak modulation depth, then ITD in the sin phase condition is less than the modulation depth in the -cos condition between 0.25 and 0.75 cycles of the modulator with the greatest difference at 0.5 cycles as shown in Fig. 1. This suggests that there should be a difference between the sin and -cos conditions for signals with durations of 0.5 cycles.

There were two potential outcomes that were anticipated regarding the improvement of FM detection with increasing signal duration. The first possibility was that the independent-samples model would predict the changes in thresholds observed across the entire range of the duration. The second was that there would be a change in performance with duration that would not be well predicted by the independent-samples model, specifically, a change in the slope of improvement with increasing duration, which is consistent with detection relying primarily on onset cues.

### B. Participants

Participants included nine young normal-hearing adults (six female; seven returned from experiment I). The seven participants that returned from experiment I were highly trained in the FM detection task, having completed at least 9 h of FM detection between six and eight months earlier. All participants completed additional training described below. Data collection was completed over the course of 1–2 sessions, lasting approximately 2 h each, with a total of 2–4 h of testing per participant. All participants provided written consent for study participation, and all procedures were approved by the university Institutional Review Board. Participants were compensated at an hourly rate.

### C. Stimuli

A total of eight conditions were tested, including all combinations of four stimulus durations (500, 250, 125, and 62.5 ms) and two modulation starting phases (sin and -cos). Testing durations shorter than 62.5 ms would require changing additional experimental parameters (i.e., 20-ms cosine-squared rise/fall window) and, therefore, was not performed. Conditions included a dichotic (FM/FM) presentation only. The standard interval consisted of an unmodulated pure tone, whereas the signal interval consisted of a pure tone with a 2-Hz modulation frequency. The frequency of the carrier was randomized on each trial, and the absolute range of frequencies that traversed over the course of a cycle of modulation was equal in both the sin and -cos starting phase conditions. The carrier frequency range and presentation level were identical to those in experiment I and experiment II.

### D. Procedures

Due to the difficult nature of the short-duration FM task, all participants began experiment III with a practice phase. Each participant completed a minimum of three practice runs at a stimulus duration of 1000 ms until thresholds were consistent with the 1000-ms thresholds in experiment I. All other procedures were identical to those in experiments I and II.

### E. Statistical analyses

Improvement in dichotic FM detection with increasing duration was analyzed with a linear mixed-effects model fit to dichotic FM detection data combined across experiments I and III. As in the other experiments, FM detection thresholds were converted to log units prior to analysis. The null hypothesis was that the independent-samples model described the improvement with increasing duration. A linear mixed-effects model was used to test for a change in the slope of improvement in threshold with increasing duration (in cycles) compared to a constant slope predicted by the independent-samples model. The full model included fixed effects for modulator starting phase, duration, change in duration (squared duration), and interactions for phase by duration and phase by squared duration. A random intercept was included for each subject. The models were fit to the residuals of the data after subtracting the prediction of the independent-samples model, which was initially computed as a line with a slope of −0.5 and an intercept of the overall mean across subjects and conditions at 500 ms (1 cycle) of −0.88 log_2_(Hz). For clarity, the reported values represent the fit to the original data with the slope and intercept of the IS model added back into the estimated parameters. A simulated likelihood ratio test was used to evaluate the significance of including each parameter in the model with a criterion of α = 0.05. The strength of the justification for additional parameters was evaluated using the Bayesian information criterion (BIC).

Data from experiment III were combined with data from the dichotic condition in experiment I for which there was not sufficient power to evaluate the predicted difference in slope relative to the independent-samples model. Each threshold obtained in both experiments was included in the analyses such that the seven participants who completed both experiments I and III provided threshold estimates in both the sin and -cos modulator starting phase at 0.125, 0.25, 0.5, 1.5, 2, 2.5 cycles, and two threshold estimates at 1 cycle. Participants who were unable to return for experiment III had thresholds in conditions reported in experiment I only, and two additional participants had thresholds from experiment III only. The random effect of subject added a single intercept value for each subject, representing their deviation from the mean performance independent of condition or duration.

### F. Results

To evaluate the hypothesis that dichotic FM detection was consistent with the use of primarily onset cues [Eq. (4)], data from experiments I and III were combined as described above. The prediction was that the improvement with increasing duration would slow as the duration increased beyond 300 ms (0.6 cycles), consistent with similar tasks relying on interaural timing cues. Linear mixed-effects modeling was used to test the improvement in the model fit, allowing for a change in the rate of improvement compared to a model with a fixed rate of improvement with increasing duration. A total of 152 FM thresholds were included in the model. The full model showed that only the fixed effects of duration and squared duration and the random effect of subject were different from zero by an amount greater than the 95% CI of the estimated parameter. Subsequent analyses included only these parameters. The model parameters are shown in Table V.

**TABLE V.** Parameter estimates for dichotic FM detection. CI, *confidence interval*; ^†^statistical comparison to −0.88 log_2_(Hz); ^‡^statistical comparison to −0.5 octaves per doubling of duration.

	*b* (95% CI)	SE	*t*	*P*
Intercept^†^	−0.90 (−1.47,0.33)	0.29	−0.07	0.94
Duration^‡^	−0.29 (−0.46,-0.12)	0.09	2.47	0.015
Duration^2^	0.25 (0.17,0.33)	0.04	6.15	<0.001
Phase	0.06 (−0.21,0.33)	0.14	0.43	0.67
Phase × duration	0.00 (−0.23,0.23)	0.12	0.03	0.98
Phase × duration^2^	−0.06 (−0.18,0.05)	0.06	−1.05	0.29
Random intercept (subject: 12 levels)	0.94 (0.62,1.42)	—	—	—
Random intercept (experiment: 2 levels)	0.00 (0.00,0.00)	—	—	—

The full model, including fixed effects of duration, squared duration, and a random effect of subject, accounted for 75.2% of the total variance based on the adjusted *R*^2^. This model has 2 free parameters for the fixed effects and a random intercept for each of 12 subjects. The null hypothesis was that the IS model would explain the improvement in detection thresholds with increasing duration. To test the improvement with the addition of the free parameters for a change in duration, a model was fit including only a random intercept for each subject. This model with no fixed effects coefficients and 12 intercept coefficients accounted for 62.2% of the variance based on the adjusted *R*^2^. To answer the question of whether adding the additional free parameters improved the model, the models were compared using a simulated likelihood ratio test with 100 simulations. The full model (log likelihood = −162.6, degrees of freedom (DF) = 4) was a significant improvement over the IS model with a random effect of subject (log likelihood = −193.0, DF = 2) with a likelihood ratio of 60.9 (*p* = 0.01). The justification of the increased model complexity was very strong based on an improvement from 396 to 345 in the BIC (Kass and Raftery, 1995). These results demonstrate that the rate of improvement with increasing duration changed as duration increased. The fact that the data were fit better by a function in which there was not a linear improvement with increasing duration, as in the IS model, is consistent with the hypothesis that cues arriving later in the signal contribute less to dichotic FM detection than the onset cues.

### G. Discussion

Dichotic FM results in periodic changes in ITD that serve as a cue for the detection of FM. In experiment III, we tested the hypothesis that onset dominance in the use of ITD cues would result in a change in the rate of improvement with increasing duration, consistent with similar tasks involving ITD cues with tonal or narrowband stimuli in which there is no strong ITD cue at the onset. Dichotic FM differs from these tasks in that the ITD cue continuously fluctuates throughout the duration of the signal. In contrast to the monaural FM thresholds evaluated in experiment II, a simple linear model (the IS model with a random intercept for each subject) fit to the dichotic data in experiment III did not explain as much of the variance as a model that allowed for a change in the rate of improvement with increasing duration. The introduction of two parameters, duration and squared duration, improved the prediction of the model sufficiently to justify the additional model complexity both in terms of a statistically significant change in the likelihood of the model prediction and a large improvement in the BIC.

Experiment I found no difference in dichotic FM detection between the sin and -cos starting modulation phase conditions. This result was consistent with the use of peak-to-peak ITD because the peak-to-peak ITD was the same for both phase conditions but the midline-to-peak (or maximum overall) ITD was different by a factor of 2. The results of experiment III were consistent with those of experiment I. Future work should address potential differences in the sensitivity to modulation in spatial location on the basis of the region of azimuth spanned by the modulated stimuli.

One potential concern with the conclusions of experiment III is that the change in the improvement with increasing signal duration was primarily due to poor detection thresholds at 0.125 cycles. The improvement from 0.125 to 0.25 cycles was much greater than the improvement predicted by the theoretical optimal integration of additional information, which is the amount predicted by the IS model of -0.5 octaves for one doubling of duration. It is possible that detection at 0.125 cycles was limited by factors other than signal duration. The null hypothesis of experiment III was that improvement for durations up to approximately 300 ms (0.6 cycles) would be consistent with the IS model, and the experiment was designed to test the hypothesis that improvement would decrease to a slope less than the slope predicted by the IS model for signals longer than that due to the use of primarily onset ITD cues. The change in slope reported here may have been due instead to the large improvement from 0.125 to 0.25 cycles, followed by steady improvement at 0.25 cycles and above consistent with the IS model. The improvement from 0.125 to 0.25 cycles was greater than would be expected from optimal integration and could reflect a breakdown of the linear systems approach at small fractions of a cycle. To address this concern, the statistical modeling was repeated, excluding thresholds obtained at 0.125 cycles. Statistical analyses were performed as described above, but parameters that were not different from zero were not included in the full model.

**FIG. 5.**
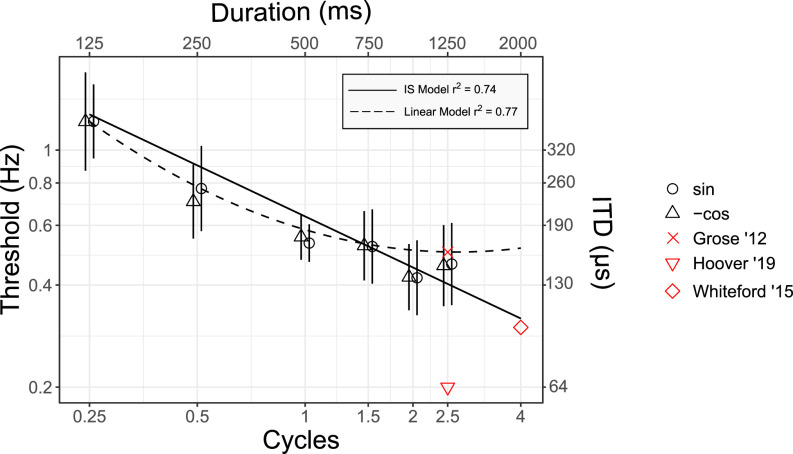
(Color online) Experiment III: Effect of short stimulus duration on dichotic FM detection, excluding data at 1/8 of a cycle. Mean dichotic FM detection thresholds are shown with standard error bars as a function of modulation cycles.

As above, the full model consisted of fixed effects of duration, squared duration, and a random effect of subject. The results are shown in Table VI and Fig. 5. The adjusted *R*^2^ of the full model was 0.768. This model was compared to the IS model, including a random effect of subject, which had an adjusted *R*^2^ of 0.745. A simulated likelihood ratio test with 100 simulations showed a significant improvement in the model prediction (likelihood ratio 13.2, *p* = 0.01). The BIC improved from 272 to 269, providing moderate justification for the additional model complexity. In summary, excluding the potentially influential data point at 0.125 cycles from the statistical analyses resulted in the same conclusion: the rate of improvement with duration in dichotic FM decreased with increasing duration, which is consistent with the use of primarily onset cues. However, support for the more complex model compared to the fixed slope of the IS model was only moderate according to the change in the BIC.

**TABLE VI.** Parameter estimates for dichotic FM detection, excluding 0.125 cycles. CI, *confidence interval*; ^†^statistical comparison to −0.88 log_2_(Hz); ^‡^statistical comparison to −0.5 octaves per doubling of duration.

	*b* (95% CI)	SE	*t*	*P*
Intercept^†^	−0.78 (−1.31,−0.25)	0.27	0.37	0.71
Duration^‡^	−0.31 (−0.42,−0.20)	0.05	3.56	<0.001
Duration^2^	0.11 (0.03,0.20)	0.04	2.77	0.006
Random intercept (subject: 12 levels)	0.03 (−0.28,0.49)	—	—	—

The results of experiment III confirmed that temporal integration had a different slope as duration increased, which is based on the moderate improvement in the variance explained by the model that allowed the rate of improvement with the duration to change. In the final model, both duration and squared duration parameters fit to the data (excluding 0.125 cycles) had a positive slope, indicating a decreasing rate of improvement with duration. This is consistent with the hypothesis that listeners rely on onsets even when there is no initial ITD present in the signal such that information arriving later in the signal is weighted less than that arriving at the beginning. One question that remains is whether there is a lower limit of modulation cycles or overall duration below which perception is limited by the total deviation in ITD as was observed for 0.125 cycles. Furthermore, it remains unclear whether the phase alignment of the modulator can influence the detection when less than the full peak-to-peak modulation depth is presented to the listener. Future work should address these questions by designing stimuli that vary parametrically in terms of the amount of binaural information available in successive portions of a frequency-modulated signal.

## V. GENERAL DISCUSSION

For monaural FM detection at low modulation rates, previous studies showed that improvement with duration was consistent with optimal integration of independent samples of the signal over time (Hartmann and Klein, 1980; Wallaert *et al.*, 2018). However, signal duration and the number of modulation cycles covaried in these studies, resulting in ambiguity regarding whether improvement relied on integration of the carrier or modulator. The present study showed that improvement with increasing signal duration depends on the number of cycles of the modulator and not on the overall signal duration. This result suggests that the modulator signal is extracted from the carrier prior to detection. Similar improvement with cycles of the modulator has been reported for monaural AM detection (Viemeister, 1979), AM rate discrimination (Lee, 1994), and AM depth discrimination tasks (Lee and Bacon, 1997). This similarity is consistent with a shared detection mechanism underlying the detection of AM and FM and the FM-to-AM conversion hypothesis, which proposes direct conversion to AM at the periphery rather than phase-locked firing to the TFS (Zwicker, 1956; Khanna and Teich, 1989). FM-to-AM conversion provides a parsimonious explanation for the dependence of monaural FM detection on the number of cycles of the modulator. However, this study cannot reject the hypothesis that FM detection relies on phase-locked firing, and can only conclude that the modulator is extracted prior to detection by some mechanism.

We hypothesized that dichotic FM detection, like other ITD tasks with sparse, tonal, or narrowband signals, relies primarily on cues contained in the onset of the stimulus. If the detection of dichotic FM is dominated by information in the onset of the stimulus, then the rate of improvement with duration should be less than that predicted by the independent-samples model. This is because useful ITD information is primarily extracted from the onsets, resulting in suboptimal use of information in the ongoing portion of the stimulus. Experiment I did not provide evidence for this hypothesis as we did not see a difference in the rate of improvement with duration between monaural and dichotic thresholds. This may have been due to the variability in dichotic FM detection thresholds, which was small in absolute terms but large relative to the low thresholds for dichotic FM. Additional data were collected at shorter durations in experiment III, improving our ability to detect a change in the rate of improvement with duration. The results from experiment III indicate that a model with additional parameters, allowing for a change in the rate of improvement, was a better fit to the dichotic data than the independent-samples model, and this remained true after eliminating the influential point at 1/8 cycle, where detection may have been limited by some unknown factor. These data suggest that dichotic FM detection relies primarily on the signal onset, similar to previous reports of ITD tasks with sparse, tonal, and narrowband stimuli (Freyman *et al.*, 1997; Freyman and Zurek, 2017) and those with fluctuating ITD (e.g., McFadden and Moffitt, 1977; Hafter *et al.*, 1979; Stecker and Hafter, 2002).

The starting phase (sin or -cos) of the modulator resulted in no differences across any of the conditions tested in experiments I and III, even for partial cycles of modulation. For monaural FM detection, this result was not surprising as there was no difference in the total number of cycles between sin and -cos starting phases. In the dichotic conditions, the different starting phases of the modulator resulted in numerous differences in the resulting waveforms. It is notable that these differences had no effect on detection, even when the duration of the signal was 62.5 ms (1/8 of a cycle of the modulator). It is clear that potential ambiguity in the implementation of the equation typically given for FM stimuli, as described above, has no effect on detection thresholds.

In the context of developing a clinically useful assay of auditory processing, we have shown that monaural and dichotic FM detection tasks are robust and efficient indices of temporal processing (Hoover *et al.*, 2019), and the results of the present study suggest that efficiency can be improved by using signals with a shorter duration. In experiment I, the difference in detection thresholds for monaural and dichotic FM for the 1250 ms duration are consistent with those reported previously for the same duration (e.g., Grose and Mamo, 2012), and the variance in thresholds for both monaural and dichotic FM detection was consistent with previous studies independent of duration with the exception of the shortest duration tested (0.125 cycles). Thus, future development of FM detection as a clinical tool may use signals with a short duration to reduce the test time and potentially improve sensitivity to impairment.

Whereas differences in monaural versus binaural temporal processing should not be surprising because of the availability of ITD cues, the differences shown here suggest that the decision to evaluate monaural or dichotic FM in a clinical setting should take into consideration the different underlying mechanisms. If FM detection relies on peripheral encoding of TFS, then the mechanism of extraction of the modulator prior to detection should be determined if FM detection is to continue to be used as an index of TFS processing ability. Future work should evaluate whether impaired temporal processing, as demonstrated using a monaural FM task, can account for dichotic thresholds or if it is possible for one and not the other to be impaired. This would allow for a greater characterization of hearing impairment as well as an improved understanding of how temporal processing relates to complex tasks such as speech intelligibility in noise.

## VI. CONCLUSIONS

The goal of the present study was to evaluate the effects of signal duration and modulator starting phase on FM detection, stimulus characteristics that differed in previous studies. There were three major conclusions of this study. (1) For monaural FM, the rate of improvement in detection thresholds with increasing signal duration is well predicted by a simple integration model as a function of the number of cycles of the modulator. (2) For both monaural and dichotic conditions, the starting phase of the modulator has no influence on the detection thresholds. Our results suggest that listeners use peak-to-peak ITD and not midline-to-peak ITD to detect dichotic FM, but the results of experiment III were ambiguous regarding the extent to which partial cycles of ITD deviation influence detection. Finally, (3) the change in the rate of improvement with increasing duration in dichotic FM detection was consistent with the hypothesis that listeners rely on onset cues to detect dichotic modulation, resulting in less efficient temporal integration than predicted by the independent-samples model. This was demonstrated by the improvement in the model with the addition of a quadratic term that allowed the rate of improvement in modulation detection to decrease with duration. The results of this study facilitate the comparison of dichotic and monaural FM detection thresholds evaluated at different durations and suggest that comparable thresholds can be obtained across a range of duration in both conditions when the rate of improvement with duration is taken into account. Future work should use a combination of behavioral, computational, and physiological methods to propose and evaluate the wide range of potential mechanisms that could underlie FM detection. Clinical research and practice can both benefit from additional studies that examine the effects of different peripheral and central temporal processing deficits on FM detection to determine the best method to index TFS perception in diagnostic and rehabilitative audiology.
